# Beyond the trends: policy considerations in psychiatric rehabilitation

**DOI:** 10.1186/2045-4015-1-25

**Published:** 2012-06-20

**Authors:** Howard H Goldman, Richard G Frank

**Affiliations:** 1 School of Medicine, University of Maryland, 1501 S Edgewood Street, Suite L, Baltimore, MD, 21227, USA; 2 Department of Health Care Policy, Harvard University, 180 Longwood Avenue, Boston, MA, 02420, USA

**Keywords:** Psychiatric rehabilitation, Mental health services, Evidence-based practices

## Abstract

Beyond the trends in the use of rehabilitative services in response to a new policy and its fiscal incentives, it is important to consider the effectiveness and quality of the services provided to individuals with mental disabilities in Israel. What is known about the outcomes of different rehabilitative services, and what is their value compared to other types of health and mental health services? Can typical health insurance be used to finance such services? These are some of the broader international questions raised by this report on the impact of a new law encouraging rehabilitation services in the community for individuals with psychiatric disabilities.

## 

Western nations have been struggling with finding ways to shift the locus of mental health care from institutions to community and to put greater efforts into rehabilitation of people with severe and persistent mental disorders. The paper “Trends in the use of rehabilitation services in the community by people with mental disabilities in Israel: the factors involved” by Hornik-Lurie, Zilber, and Lerner [1] provides a detailed picture of the pattern of response to a major piece of mental health and social welfare legislation in Israel. The study documents the increases in utilization for specific rehabilitation services and describes the population of service users who responded to the new entitlement and incentives in the reform legislation.

The new policy provided an entitlement to a market basket of rehabilitation services for qualified individuals disabled by a mental illness. Champions of the reform, Uri Aviram and his colleagues [2], regard the legislation and reform policy as important principally because the newly implemented mental health services in Israel follows an international standard of care, such as the recommendations of a presidential commission in the United States [3] and a standard textbook of psychiatric rehabilitation [4].

Apart from demonstrating the expected response of a population to the entitlement and incentives of a new reform policy, what is the larger significance of the paper by Hornik-Lurie and her colleagues? What might the international mental health field learn and consider in the findings and discussion of the impact of Israel’s Rehabilitation in the Community of Persons with Mental Disabilities Law?

In our view there are three major implications of their work suggesting further consideration. 1. What is known about the effectiveness and likely outcomes of the specific services used over the past decade in response to the new law and reformist rehabilitation policy? 2. Which of the specific services should be encouraged within the mental health system, and which are cost-effective even when compared to other potential investments in services within the health care system and more broadly within the social welfare system? 3. Are these newly implemented rehabilitation services appropriate for inclusion in a benefit package for health care insurance?

## What is known about effectiveness?

Hornik-Lurie and colleagues report that following implementation of the law, a wide array of psychiatric rehabilitation services were authorized and provided. Some of the services are supported by the results from a number of well-designed studies of effectiveness and others are not. All of them are offered as progressive alternatives to inpatient care, and while in general this is to be favored in a modern mental health service system, some of the services provided are more likely than others to achieve good health and social welfare outcomes. Their clinical and quality of life objectives vary considerably as well, ranging from recovery of function to participation and inclusion in the wider society. For example, while there is little evidence to support one form of residential alternative to long-term hospitalization over another, there is considerable evidence to favor supported employment over various other approaches to occupational rehabilitation in terms of return to some level of workforce participation [5].

## How do the rehabilitation services compare to other health and human services?

Although there is evidence to support implementing some of the psychiatric rehabilitation approaches over other approaches to rehabilitation, there is less evidence to support psychiatric rehabilitation over other types of behavioral health services. While directors of mental health services may have ample empirical evidence that they should implement supported employment rather than sheltered workshops or other vocational services, they have less evidence to guide them toward decisions about allocating resources to rehabilitation rather than preventive services or early case identification and assertive treatment engagement. Supported employment promotes participation in competitive employment for individuals with severe mental impairments, but it rarely returns individuals to fulltime worker status. This is a remarkable accomplishment towards objectives of social inclusion and participation by recovering individuals with a history of mental illness, but it is not a solution to longstanding problems of poverty among disabled individuals. Other interventions may prove even more remarkable in achieving recovery objectives and reversing the untoward effects of mental illness. The implementation of an array of rehabilitative services encourages us to believe that we can alter the course of mental health services delivery, and it also raises questions about the desired outcomes and the effectiveness of various interventions.

## Is traditional health insurance likely to cover long-term psychiatric rehabilitation services?

The success of policy implementation in psychiatric rehabilitation leads us to think about the appropriate approach to financing such services. In Israel the reform law created an entitlement to health and social insurance benefits. In the United States, the health reforms ushered in by the Affordable Care Act (ACA) similarly mandated coverage for rehabilitation services. In contrast to the array of services funded in Israel, rehabilitation services covered by health insurance in the U.S. typically are restricted to short-term interventions following acute illnesses or accidents. For example, insurance covers a short course of cardiac rehabilitation following a myocardial infarction or physical rehabilitation following a fracture or back injury. It might fund some psychiatric rehabilitation following an acute admission to hospital for a psychotic illness, but it would not cover services of indefinite duration for chronic conditions. The means-tested Medicaid program that serves the poor, the disabled, and frail elders does cover some supportive services related to psychiatric rehabilitation, such as those associated with supported employment, but the core elements of such vocational interventions such as job finding and job coaching are rarely covered by any health insurance schemes and are not likely to be covered under the ACA.

## Conclusion

The paper by Hornik-Lurie and her colleagues reveals much about the implementation of an important progressive law in Israel. It provides encouragement for similar reforms around the world. It also raises many more questions about how one practically crafts entitlement programs through the application of studies of effectiveness and cost-benefit analysis. We recognize that in many cases research can provide a useful guide, but in others we must make a bet and follow that up with careful evaluation. Beyond setting out the contents of the service bundle in a health insurance scheme, it will be essential to develop a policy for paying for services in a fashion that encourages both sound clinical judgments and efficient use of public resources.

## Misc

Commentary on the paper by Hornik-Lurie, Zilber, Lerner: Trends in the use of rehabilitation services in the community by people with mental disabilities in Israel; the factors involved.

## Authors' information

Howard H Goldman is a mental health services and policy researcher. He specializes in studies of financing policy and evaluations of demonstration programs implementing evidence-based services. Dr. Goldman served as the Senior Scientific Editor of *Mental health: a report of the Surgeon General* and as a consultant to the President’s New Freedom Commission on Mental Health. He is the editor of *Psychiatric Services*, a research journal published monthly by the American Psychiatric Association.

Richard G Frank is the Margaret T Morris Professor of Health Economics at Harvard University Medical School. He is also affiliated with the National Bureau of Economic Research. Dr. Frank conducts research on the economics of mental health, disability, and aging. Between 2009 and 2011 Professor Frank served as Deputy Assistant Secretary in the U.S. Department of Health and Human Services.

Dr. Goldman has no commercial conflicts of interest.

Dr. Frank has no commercial conflicts of interest
